# Oxidative Stress in Takotsubo Syndrome: Insights into Extracellular Vesicles and Their Potential Clinical Relevance

**DOI:** 10.3390/antiox15030302

**Published:** 2026-02-27

**Authors:** Rosa Ciullo, Saveria Femminò, Maria Felice Brizzi, Pasquale Pagliaro, Claudia Penna

**Affiliations:** 1 Department of Clinical and Biological Science, University of Torino, Regione Gonzole 10, 10043 Orbassano, Italy; rosa.ciullo@unito.it (R.C.); pasquale.pagliaro@unito.it (P.P.); claudia.penna@unito.it (C.P.); 2 Department of Medical Science, University of Torino, Corso Dogliotti 14, 10126 Turin, Italy; mariafelice.brizzi@unito.it; 3 National Institute for Cardiovascular Research (INRC), 40126 Bologna, Italy

**Keywords:** biomarkers, heart failure, microRNAs, oxidative stress

## Abstract

Takotsubo syndrome (TTS) is an acute and reversible form of heart failure characterized by transient left ventricular dysfunction, typically triggered by acute stress stimuli. TTS, also referred to as “stress cardiomyopathy”, may paradoxically be triggered not only by negative stressors but also by intense positive emotional experiences. Interestingly, TTS was sharply incremented during and following the COVID-19 pandemic. Despite increased clinical recognition, reliable biomarkers for early diagnosis and prognosis remains limited. Oxidative stress is increasingly recognized as a key mechanism in TTS, acting downstream of sympathetic overactivation, thus contributing to myocardial stunning, endothelial dysfunction, and inflammation. In this context, extracellular vesicles (EVs) have emerged as key mediators of intercellular communication and as potential circulating biomarkers, as they reflect the molecular state of their cells of origin. In this review, we summarize the current diagnostic approaches for TTS, including the InterTAK Diagnostic Score, imaging gold standards, and emerging biomarkers such as circulating miRNAs and EV cargo associated with TTS. Furthermore, we critically examine the mechanistic interplay between oxidative stress and EVs in TTS, highlighting translational perspectives and future directions for integrating EV-based biomarkers into personalized clinical management.

## 1. Introduction

First described in Japan in 1990 by Dr Hiraku Sato, Takotsubo syndrome (TTS) is a cardiac reversible pathological condition, also known as “stress cardiomyopathy” or “broken heart disease” [1].

TTS displays distinctive clinical features that differentiate it from other acute cardiac conditions. Accounting for up to 2% of acute coronary syndrome (ACS) cases in the absence of obstructive coronary artery disease or plaque rupture, TTS is characterized by transient left ventricular dysfunction and is frequently triggered by emotional or physical stress [2]. Surprisingly, TTS can also be triggered by positive emotional experiences, a condition called “happy heart syndrome”, as opposed to the classic term “broken heart syndrome”, which refers to negative emotion causes [3].

Notably, the prevalence of this pathological condition sharply increased during and following COVID-19 pandemic, with reported rates of 7.75% compared to 1.5–1.8% in earlier period, raising awareness of this disorder [4]. Interestingly, while TTS predominantly affects women, especially postmenopausal women, men tend to experience a worse prognosis with higher rates of both in-hospital and long-term mortality [5].

Although TTS is known to be a reversible condition, approximately one-fifth of patients are at risk of serious adverse in-hospital events that occur as a result of hemodynamic and electrical instability [6]. Specifically, acute heart failure, left ventricular outflow tract obstruction (LVOTO), mitral regurgitation, and cardiogenic shock are the most common, but less frequent events, such as ventricular arrhythmias and intraventricular thrombus formation, may also occur [7].

Despite emerging interest in defining the disease process of TTS, coexisting medical conditions and their effect on patient outcomes remain unclear. Psychiatric comorbidities have been reported to have a prevalence of approximately 25% in TTS patients and are associated with a significantly higher risk of TTS relapse at follow-up [8]. Neurological disorders also represent a risk factor and identify a subgroup of TTS at high risk for an enhanced mortality rate in both the short and long term [9].

Moreover, a common comorbidity associated with TTS is cancer. Previous studies have highlighted a high incidence of malignancies in patients diagnosed with TTS, and recent observational studies have also reported a higher mortality rate in patients with TTS complicated with cancer compared to those without cancer [10,11,12]. Interestingly, a so-called “diabetes paradox” has been described in TTS, with several observational studies reporting a lower prevalence of diabetes mellitus among affected patients [13,14]. It has been hypothesized that advanced diabetes and its associated autonomic neuropathy may attenuate sympathetic nervous system-induced stunning and thereby potentially reduce susceptibility to TTS [15].

Other known risk factors that have been associated with TTS include, for example, obesity, present in 17% of patients, hypertension in 54%, dyslipidemia in 32%, and smoking in 22% [16].

Although significant progress has been made in recent years in understanding this clinical entity, TTS continues to challenge our understanding, and its underlying pathophysiology remains unclear, with diagnosis often relying on exclusion [17]. The most widely accepted hypothesis proposes that a surge in catecholamines and sympathetic overactivation leads to myocardial injury, microvascular dysfunction, and myocardial stunning [18]. More recently, the interplay of additional mechanisms has been brought into focus, including endothelial dysfunction, platelet activation, inflammatory responses, and oxidative stress [18,19].

Oxidative stress is defined as an imbalance between excessive reactive oxygen species (ROS) generation and the efficiency of antioxidant defense mechanisms to counteract them [20]. Excessive ROS production can damage proteins, lipids, and nucleic acids, thereby disrupting cellular homeostasis and leading to mitochondrial dysfunction, impaired excitation-contraction coupling, and cardiomyocyte death [21,22].

While oxidative stress is known to play a role in several acute and chronic cardiovascular diseases (CVDs), its contribution to TTS is only beginning to be explored [23,24,25]. Since, in the context of TTS, one of primary triggers is thought to be a surge of catecholamines, recent evidence highlights the role of catecholamine oxidation, which generates ROS and reactive metabolites that can directly injure cardiomyocytes [26].

Collectively, this evidence highlights the role of oxidative stress pathways in TTS. Extracellular vesicles (EVs) are small, rounded, double-membrane particles released by cells under both physiological and pathological stress conditions and play a pivotal role in cell-to-cell and cell-to-environment communication through their cargo. Investigating the involvement of EVs in TTS is, therefore, of particular relevance [27,28]. Notably, EVs have been already identified as biomarkers for several CVDs [29,30,31]. In this scenario, the present review aims to provide an overview which integrates existing evidence to propose mechanistic links between oxidative stress pathways, EVs, and TTS while highlighting potential diagnostic and prognostic biomarkers and future research perspectives. Finally, we highlight the current diagnostic approaches to TTS, including electrocardiography, the InterTAK Diagnostic Score, endomyocardial biopsy, circulating inflammatory cytokines, and major imaging techniques.

## 2. Pathophysiology of Takotsubo Syndrome

The TTS pathophysiology is not fully understood, but compelling evidence identifies sympathetic activation as a key pathogenic driver. Specifically, in TTS patients, physical or emotional stress events lead to catecholamines secretion by adrenal medulla [32]. Sustained sympathetic nervous system hyperactivity induces an abnormal elevation in circulating catecholamines (epinephrine and norepinephrine), achieving levels approaching a threefold increase compared with patients with acute myocardial infarction [33,34]. Furthermore, as demonstrated by several authors [35,36], systemic infusion of catecholamines and β-adrenergic receptor agonists can reproduce a stress-induced cardiomyopathy in susceptible individuals, thereby supporting the link between catecholamine release, cardiac stress, and TTS. In contrast, reduced parasympathetic nervous system activity has been observed. At the myocardial level, elevated concentrations of myocardium-derived norepinephrine have been detected in patients with TTS, likely contributing to increased vulnerability to myocardial stunning and contractile dysfunction [37]. Excess myocardial catecholamines overwhelm β-adrenergic signaling pathways within cardiomyocytes. This process results in altered intracellular signal transduction, calcium overload, and impaired excitation–contraction coupling, particularly in apical segments where adrenoceptor density is highest [38]. This catecholamine-mediated dysregulation exerts multiple deleterious effects on the heart through both vascular and cellular mechanisms. In particular, the marked elevation of circulating and locally released catecholamines leads to regional wall motion alterations, reduction in contractile performance, and transient left ventricular ballooning, despite the absence of obstructive coronary artery disease [39]. At the epicardial level, excessive sympathetic stimulation may trigger diffuse, multi-vessel myocardial spasm, leading to transient ischemia that is not confined to a single coronary territory [40]. Concomitantly, catecholamine surges impair coronary microcirculatory function by inducing endothelial dysfunction, microvascular constriction, and reduced coronary flow reserve, thereby further compromising myocardial perfusion [41]. Thus, both epicardial vessels and the microcirculation alterations are frequently observed in TTS patients. However, it remains unclear whether these dysfunctions represent a primary trigger or a downstream consequence of the acute event. When microvascular impairment is documented during the acute phase, it appears to be reversible, with normalization paralleling the recovery of left ventricular performance [42]. This temporal association supports the concept that coronary microcirculatory disturbances, together with their related manifestations, are closely intertwined with the pathophysiological evolution of TTS rather than being incidental findings. At a cellular level, direct catecholamine toxicity on cardiomyocytes results from exaggerated β-adrenergic receptor activation, promoting intracellular calcium overload, oxidative stress, and mitochondrial dysfunction, which collectively contribute to myocardial stunning and contractile failure [43,44,45]. Paradoxically, this intense adrenergic stimulation may also activate compensatory myocardial survival pathways, including stress-response cascades, which limit irreversible injury and help explain the typically transient and reversible nature of left ventricular dysfunction observed in TTS [46]. Overall, these mechanisms provide a conceivable pathophysiological link between elevated catecholamine levels and the acute cardiac impairment seen in patients with TTS.

## 3. Oxidative Stress in Takotsubo Syndrome

Oxidative stress has emerged as a pivotal mechanism in the development of TTS, acting as a downstream effector of neurohumoral activation and sympathetic overdrive. As reported above, catecholamines stimulate β-adrenergic receptors, leading to excessive intracellular calcium influx and heightened mitochondrial workload. Under these conditions, mitochondrial electron transport becomes inefficient, favoring electron leakage and the generation of ROS [47]. Simultaneously, catecholamines undergo auto-oxidation and enzymatic metabolism via monoamine oxidase, further contributing to hydrogen peroxide and superoxide production. These processes result in a profound redox imbalance, particularly in regions of the left ventricle, exhibiting the greatest functional impairment [48]. Directly, oxidative stress contributes to myocardial stunning by modifying contractile proteins and key signaling pathways involved in excitation–contraction coupling. ROS-mediated oxidation of sarcoplasmic reticulum calcium-handling proteins, such as sarco/endoplasmic reticulum Ca^2+^-ATPase 2a (SERCA2a) and ryanodine receptors [49]. In parallel, oxidative stress activates stress-sensitive kinases and transcription factors, including mitogen-activated protein kinases (MAPKs) and nuclear factor kappa-light-chain-enhancer of activated B cells (NF-κB), which further amplify inflammatory and apoptotic signaling cascades [50]. Consistently, the inflammatory state has been confirmed in a clinical study, in which a cohort of postmenopausal women with TTS exhibited elevated C-reactive protein (CRP) levels that were associated with impairment of left ventricular ejection fraction (LVEF) [51]. Notably, CRP levels did not peak at hospital admission, but increased over the subsequent days. This suggests that the inflammatory state is unlikely to represent a primary trigger of TTS and may instead reflect a downstream consequence or an associated pathophysiological mechanism.

The coronary microvasculature appears particularly vulnerable to oxidative injury in TTS. Endothelial dysfunction driven by ROS may explain the mismatch between relatively preserved epicardial coronary arteries and profound regional myocardial dysfunction. In fact, it has been reported that endothelial nitric oxide synthase (eNOS) activity is dysregulated in TTS patients [52]. In physiological conditions, nitric oxide (NO) is produced by eNOS, by maintaining vascular integrity and homeostasis. Since the cofactor of eNOS, named tetrahydrobiopterin (BH4), is depleted in TTS patients, eNOS preferentially generates ROS rather than NO, resulting in ROS accumulation and further exacerbation of the myocardial damage [53].

Mitochondrial dysfunction is likewise a hallmark event of oxidative stress and contributes to dysregulated myocardial metabolism. This process results in mitochondrial injury, reduced ATP production, and disruption of cellular homeostasis. These alterations ultimately impair cardiomyocyte viability and function, further amplifying myocardial damage in these patients [54]. As reported by Wu et al., in a reproduced mouse model of TTS induced by isoproterenol administration, the Hippo signaling pathway was identified as one of the key mechanisms underlying mitochondrial dysfunction [55]. In this context, the activation of the Hippo pathway is associated with YAP-TEAD1 suppression, thus leading to structural and functional alterations in cardiac mitochondria.

**Figure 1:**
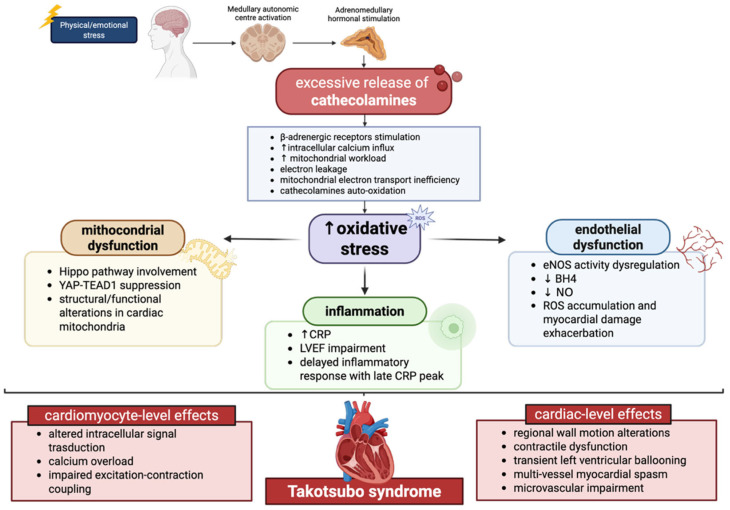
Schematic illustration of the role and effects of oxidative stress in TTS pathophysiology. In TTS patients, physical or emotional stress events triggers excessive catecholamines secretion from the adrenal medulla. Catecholamines stimulate β-adrenergic receptors, resulting in increased intracellular calcium influx and enhanced mitochondrial workload. Under these conditions, mitochondrial electron transport becomes inefficient, promoting electron leakage and the generation of ROS. The resulting redox imbalance and oxidative stress promote mitochondrial dysfunction with activation of the Hippo pathway associated with YAP-TEAD1 suppression, inflammatory responses with elevated CRP levels, and endothelial dysfunction associated with eNOS dysregulation. Altogether, this catecholamine-mediated dysregulation exerts multiple detrimental effects on the heart through both vascular and cellular mechanisms, ultimately contributing to the development of Takotsubo syndrome. BH4: Tetrahydrobiopterin; CRP: C-reactive protein; LVEF: left ventricular ejection fraction NO: nitric oxide; ROS: reactive oxygen species; ↑: increase; ↓: decrease.

Importantly, oxidative stress in TTS is not merely a marker of injury but may actively shape disease reversibility. The transient nature of left ventricular dysfunction suggests that antioxidant and mitochondrial repair mechanisms are eventually re-engaged, allowing recovery of myocardial function [47]. However, in susceptible individuals, such as postmenopausal women, this redox imbalance may be more pronounced, contributing to disease onset and severity (Figure 1).

## 4. Diagnosis of Takotsubo Syndrome

Given the lack of specific models enabling the unequivocal identification of TTS, the diagnosis relies on a diagnostic workup that follows recommendations resulting from the interpretation of limited data from currently available clinical trials and the experience of international TTS experts [40].

In addition to traditional diagnostic techniques, such as electrocardiogram (ECG), imaging techniques and biomarkers, already known in clinical practice, are also being used [40]. Increasing attention is given toward the discovery of new potential candidates, including EVs and microRNAs (miRNAs). These efforts aim to enable early, precise, and non-invasive diagnosis and to provide more detailed prognostic information on a syndrome often characterized by an uncertain clinical course.

### 4.1. Electrocardiogram

In most patients with TTS, the initial ECG is abnormal, displaying ischemic ST-segment elevation, T-wave inversion, or both [40].

Actually, TTS is associated with a dynamic and evolving pattern of ECG abnormalities that may mimic ACS. In the acute phase, ST-segment elevation is frequently observed, particularly in the precordial leads, although it is usually less pronounced and less localized than in STEMI. In some patients, ST-segment depression or non-specific ST–T changes may also occur. As the disease evolves, deep and diffuse T-wave inversions typically develop, often accompanied by QT interval prolongation, reflecting myocardial repolarization abnormalities. QT prolongation may predispose individuals to ventricular arrhythmias, including torsades de pointes, although malignant arrhythmias are relatively uncommon. Additional ECG findings may include pathological Q waves, low QRS voltage, and transient conduction abnormalities, such as atrioventricular block or bundle branch block. Importantly, ECG changes in TTS are usually reversible and tend to normalize over days to weeks, paralleling the recovery of left ventricular function [40].

In particular, in the InterTAK Registry, the ECG abnormalities were distributed as follows: ST-segment elevation was present in 44%, ST-segment depression in 8%, T-wave inversion in 41%, and left bundle branch block in 5% [7]. Moreover, similarly to ACS, the ECG exhibits dynamic temporal evolution. Both early and later ECG findings are influenced by multiple factors, including the geographic pattern of left ventricular (LV) ballooning, presence or absence of right ventricular (RV) ballooning, the interval time between symptom onset and clinical evaluation, presence of myocardial edema, and recovery rate of myocardial cellular function [40].

### 4.2. InterTAK Diagnostic Score

The InterTAK Diagnostic Score was developed by the International Takotsubo Registry to provide clinicians with a model for estimating the presence of TTS with high sensitivity, which is able to distinguish TTS from ACS [56] with high specificity.

The InterTAK Diagnostic Score takes into account seven different parameters: female sex, emotional trigger, physical trigger, absence of ST-segment depression (except in lead aVR), psychiatric disorders, neurologic disorders, and QTc prolongation [56]. Notably, these parameters can be easily obtained in the emergency department and do not require an imaging modality. All these variables are ranked according to their diagnostic importance and, based on the cross-referenced result, a maximum attainable score of 100 points is assigned [56]. In patients with non-ST-segment elevation, the InterTAK Diagnostic Score can be considered. While a low to intermediate probability of TTS is suggested in the presence of an InterTAK Score ≤ 70 points, a score ≥ 70 indicates a high probability for TTS diagnosis [40].

### 4.3. Endomyocardial Biopsy

Endomyocardial biopsy represents an important diagnostic tool when differentiating TTS from myocarditis, as these conditions can share similar clinical and instrumental findings [57]. Both may present with acute chest discomfort and reversible left ventricular dysfunction, making the differential diagnosis challenging. In carefully selected patients, histopathological analysis obtained through endomyocardial biopsy can provide decisive evidence, allowing confirmation or exclusion of myocardial inflammation consistent with myocarditis [58].

The presence of acute myocarditis does not necessarily rule out TTS. On the contrary, in some patients, myocardial inflammatory changes may represent a relevant histopathological component of the condition itself, particularly in the context of catecholamine-mediated myocardial injury, sometimes referred to as “catecholamine myocarditis”. In this setting, inflammatory findings may reflect the myocardial response to excessive adrenergic stimulation rather than a primary infectious process [59].

Overall, endomyocardial biopsy should be considered in selected cases to clarify the underlying pathology and to appropriately distinguish between primary myocarditis and TTS with inflammatory features.

### 4.4. Circulating Inflammatory Cytokines

Beyond endomyocardial biopsy, additional laboratory approaches may support the exclusion of myocarditis in patients with suspected TTS. In particular, the assessment of circulating inflammatory cytokines can provide useful information about the presence and extent of myocardial inflammation [60]. An excessive systemic inflammatory response may promote elevated circulating levels of tumor necrosis factor-α, IL-6, IL-1β, and catecholamines, potentially contributing to the onset of TTS. Consistently, high levels of cytokines, such as those observed in COVID-19 patients, may induce direct catecholamine-mediated myocardial toxicity, thereby facilitating the development of TTS [61]. Since myocarditis is typically associated with a systemic and myocardial inflammatory response, the evaluation of specific cytokine profiles may represent a complementary tool in the differential diagnosis between primary inflammatory myocardial injury and TTS.

### 4.5. Imaging Techniques

Cardiac imaging techniques include both invasive and non-invasive strategies.

#### 4.5.1. Coronary Angiography and Left Ventriculography

Although non-invasive imaging modalities are useful in the diagnostic workup, coronary angiography and left ventriculography are considered to be the gold standards for the final evaluation of the suspected TTS and differentiation from ACS [62]. These procedures are pivotal for excluding obstructive coronary disease and for revealing the wall motion abnormalities characteristic of TTS [40]. Most patients with TTS exhibit normal coronary arteries or only mild atherosclerotic changes in contrast to acute myocardial infarction, which is typically associated with significant blockages [62]. Left ventriculography, often performed alongside coronary angiography, commonly demonstrated the classic apical ballooning pattern, although midventricular, basal, and focal variants have also been described [40].

#### 4.5.2. Echocardiography

The main TTS variants can be identified using echocardiography, which is the most widely used imaging tool to assess LV dysfunction, allowing the identification of regional wall motion abnormalities that extend beyond a single coronary territory. Importantly, echocardiography also plays a crucial role in the detection of all acute TTS complications, including LV outflow tract obstruction, mitral regurgitation, thrombus formation, and ventricular rupture [63].

Echocardiography reveals characteristic and typically reversible patterns of left ventricular wall motion abnormalities. The most common presentation is apical ballooning, characterized by hypokinesia or akinesia of the apical and mid-ventricular segments with preserved or hypercontractile basal segments. However, atypical variants, including mid-ventricular, basal (reverse), focal, and global forms, are increasingly recognized [64]. Left ventricular systolic dysfunction is usually transient and may be associated with dynamic LVOTO, mitral regurgitation, and elevated filling pressures, particularly in the acute phase. Right ventricular involvement can also occur and has been associated with a more severe clinical course [65]. Additional echocardiographic findings may include increased myocardial echogenicity, reduced global longitudinal strain extending beyond a single coronary territory, and intracavitary thrombus formation in cases with severe apical akinesia. Importantly, echocardiographic abnormalities in TTS generally resolve within days to weeks, paralleling clinical improvement and recovery of ventricular function.

#### 4.5.3. Cardiac Magnetic Resonance Imaging

Cardiac magnetic resonance imaging (cardiac MRI) cannot be used easily in the acute setting of TTS but has become a cornerstone in the subacute evaluation of TTS [62]. Cardiac MRI allows precise evaluation of RV and LV function, assessment of additional complications, and high-resolution characterization of myocardial tissue, such as edema, inflammation, and necrosis/fibrosis [66]. The combination of myocardial edema and the absence of late gadolinium enhancement (LGE) is pivotal to the cardiac MRI-based diagnosis of TTS, reflecting the lack of irreversible myocardial injury. In particular, cardiac MRI with gadolinium contrast administration is useful in the differential diagnosis of acute myocardial infarction and myocarditis [62]. It is also considered the gold standard for follow-up to confirm reversibility within 3–6 months, the hallmark of TTS [67].

#### 4.5.4. Cardiac Computed Tomography

Additional imaging techniques are also employed in the clinical evaluation of TTS patients. These include cardiac computed tomography (CT), which can be considered a non-invasive alternative for providing information on both coronary artery anatomy and regional LV contraction [68].

#### 4.5.5. Nuclear Imaging

Moreover, nuclear imaging techniques, including single-photon emission computed tomography (SPECT) and positron emission tomography (PET), provide semi-quantitative and quantitative measurements, respectively, enabling the assessment of myocardial perfusion, metabolism, and innervation in TTS. In addition, sympathetic innervation imaging allows assessment of cardiac autonomic dysfunction, a key pathophysiological feature of TTS [40]. Table 1 summarizes all the diagnostic techniques for TTS described above.

### 4.6. Biomarkers

**Figure 2:**
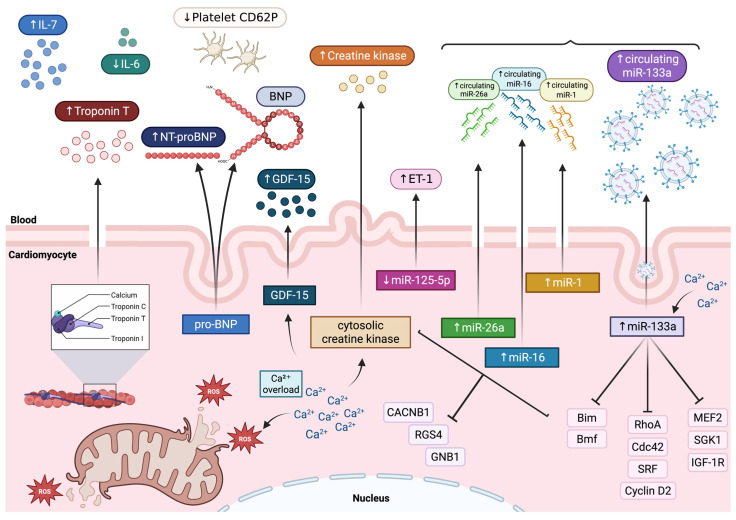
Schematic illustration of proposed mechanisms underlying validated and emerging biomarkers release in TTS. Exaggerated β-adrenergic receptor activation induced by catecholamine surge promotes intracellular calcium overload, increased wall stress, oxidative stress, and mitochondrial dysfunction in cardiomyocytes. Validated biomarkers of myocardial injury include markedly increased plasma levels of cardiac troponin and NT-proBNP plasma levels, whereas creatine kinase shows only a mild elevation. Emerging biomarkers include reduced platelet CD62P expression and low plasma levels of IL-6, but increased circulating levels of IL-7 and GDF-15. Circulating miR-133a derives mainly from EVs, and its increase significantly correlates with serum cardiac troponin T levels. Additionally, a signature of four circulating miRNAs has been reported, including miR-16, miR-26a, miR-1, and miR-133a. Functionally, miR-133a inhibits pro-apoptotic genes such as Bim and Bmf and modulates cardiac hypertrophic signaling by targeting RhoA, Cdc42, SRF, and cyclin D2. Other targets include MEF2, as well as SGK1 and IGF-1R. Overexpression of miR-16 and miR-26a, in turn, downregulates CACNB1, RGS4, and GNB1. Finally, endothelin-1-regulating miR-125a-5p shows a tendency toward downregulation in parallel with a robust increase in plasma ET-1 levels. BNP: B-type natriuretic peptide; CACNB1: L-type calcium channel Cavβ subunit; cyclin D2; EVs: extracellular vesicles; ET-1: endothelin-1; GDF-15: growth/differentiation factor 15; GNB1: G-protein subunit Gβ; IGF-1R: insulin-like growth factor-1 receptor; MEF2: myocyte enhancer factor 2; miRNAs: microRNAs; NT-proBNP: N-terminal pro-B-type natriuretic peptide; pro-BNP: pro-B-type natriuretic peptide; RGS4: regulator of G-protein signaling 4; ROS: reactive oxygen species; SGK1: serum- and glucocorticoid-responsive kinase 1; SRF: serum response factor; ↑: increase; ↓: decrease.

Cardiac biomarkers display a crucial role in situations where TTS diagnosis is unclear. In fact, TTS shows a biochemical profile that distinguishes it from ACS (Table 2 and Figure 2).

On admission, troponin levels are elevated in 87% of patients but mean levels overlap those measured in ACS patients [7]. However, the situation dramatically changes during hospitalization with an increase by a factor of 1.8 in TTS patients, compared to 6 in ACS patients [69]. Couch et al., in a meta-analysis including 27 studies, confirm that troponin levels are significantly different in TTS and ACS, with a disparity of approximately 75 times the upper limit of normal in ACS [70]. Furthermore, Templin et al. [7] highlight the association between a first troponin measurement greater than 10 times the upper limit of normal level or a left ventricular ejection fraction of less than 45% and a higher incidence of combined end point. The product of peak troponin I (TnI) levels and echocardiographically derived left ventricular ejection fraction (≥250) has recently been used as an index to differentiate TTS from ST-segment elevation myocardial infarction (STEMI), with sensitivity of 95% and specificity of 87% [71].

Typically, creatine kinase displays only a slight increase [7].

A total of 82.9% of the patients show increased levels of brain natriuretic peptide (BNP) on admission, significantly higher than ACS patients, and elevated plasma levels of N-terminal prohormone of brain natriuretic peptide (NT-proBNP), peaking approximately 24–48 h after symptom onset [7,40]. Frequently, BNP and NT-proBNP reach extremely high concentrations that closely correlate with the extent of ventricular wall motion abnormality [18]. According to existing literature, natriuretic peptides have greater diagnostic utility compared to troponins and should be measured in all TTS-suspected cases [18].

Furthermore, the serum copeptin to NT-proBNP ratio has been proposed as an additional useful biomarker in non-invasive differentiation since copeptin levels are significantly lower in TTS compared to STEMI [72]. Likewise, the ratios of NT-proBNP to markers of myocardial injury, including myoglobin and troponin T, reliably differentiate TTS from both STEMI and non-STEMI (NSTEMI) [73]. Budnik et al. have further demonstrated that NT-proBNP-based ratios, particularly NT-proBNP/TnI, NT-proBNP/creatine kinase-myocardial band (CK-MB) mass, and NT-proBNP/ejection fraction (EF), are significantly higher in TTS compared with STEMI, with the NT-proBNP/TnI ratio showing the highest diagnostic accuracy and providing a useful tool for early discrimination [74].

Other candidate biomarkers have been proposed, including platelet CD62P expression and IL-6 plasma levels, which are significantly lower, and plasma IL-7 level, which is significantly higher in patients with TTS compared to those with myocardial infarction [75]. Nevertheless, groups displayed small differences and were unlikely to be of diagnostic utility [40].

Additionally, patients with Takotsubo cardiomyopathy exhibit markedly elevated but transient growth/differentiation factor 15 (GDF-15) levels, with especially high concentrations in cases of biventricular ballooning, and admission levels strongly predict adverse clinical outcomes [76].

#### miRNA

MiRNAs are an endogenous subtype of small non-coding RNAs, which are approximately 22 nucleotides in length and are important post-transcription regulators of genes [77]. miRNAs have gained attention as potential surrogate markers for the early and accurate diagnosis of cardiovascular disease and for predicting medium- and long-term prognosis [78]. Furthermore, combining miRNAs with traditional biomarkers can improve risk stratification and long-term prognosis [78]. Indeed, miRNAs possess unique properties, including cellular or tissue specificity, stability in serum or plasma, resistance to degradative factors such as freeze–thaw cycles or enzymes in the blood, and rapid release kinetics.

Kuwabara et al. [79] demonstrated that the serum levels of miR-133a sharply increase in patients with Takotsubo cardiomyopathy. miR-133a levels significantly correlate with the cardiac troponin T level in serum, suggesting that miR-133a level indicates myocardial damage. Furthermore, miR-133a release into circulation, particularly via exosomes, correlated with the onset of myocardial damage in the TTS subgroup [79]. These findings suggest that circulating miR-133a can be used as a sensitive biomarker for cardiomyocyte death, and it may have functions in cardiovascular diseases [79].

On the other hand, Jaguszewski et al. [80] first described a signature of four circulating miRNAs, including miR-16, miR-26a, miR-1, and miR-133a, which can robustly distinguish TTS from STEMI with a sensitivity and specificity of 96.77% and 70.37%, respectively. Moreover, endothelin-1-regulating miR-125a-5p showed a tendency toward downregulation in parallel with a robust increase in plasma endothelin-1 levels in TTS patients compared with healthy subjects [80].

Notably, Toro et al. [81] showed that miR-16-5p is responsible for endoplasmic reticulum stress and oxidative stress in cardiac cells, suggesting that its inhibition may represent a potential therapeutic strategy to protect the heart against stress-induced injury. In fact, miR-16-5p is able to significantly downregulate the expression of relevant antioxidant genes superoxide dismutase (SOD), catalase (CAT), and glutathione peroxidase (GPx) in cardiomyoblast cells, confirming its involvement in promoting oxidative stress [81].

Consequently, Couch et al. [82] further investigated whether miR-16 and miR-26a had a causal relation to TTS or simply represented catecholamine activation/damage using a rodent model. The overexpression of miR-16 and miR-26a enhances susceptibility to adrenaline-induced apical dysfunction, reproducing the distinctive contractility abnormalities of TTS [82]. These microRNAs selectively depress contractility with loss of adrenergic responsiveness in apical cardiomyocytes while enhancing the initial inotropic response in basal cells, effects reproduced in human cardiomyocytes. Mechanistically, Couch et al. [82] demonstrated that these changes are mediated by downregulation of key calcium- and G-protein-signaling components: L-type calcium channel Cavβ subunit (CACNB1), regulator of G-protein signaling 4 (RGS4), and G-protein subunit Gβ (GNB1). This supports the hypothesis of neuro-cardiogenic mechanisms involving the central nervous system and dysregulation of stress-axis, since miR-16 and miR-26a are well known for their involvement in stress, depression, and anxiety pathways [83]. Furthermore, these miRNAs could represent a molecular link between previous exposure to stress and increased vulnerability to TTS [82,83].

**Table 2:** Diagnostically relevant biomarkers, including both clinically established biomarkers in TTS and emerging biomarkers that have shown promise as diagnostic tools but require further study.

Biomarker	Pathophysiological Category	Typical Pattern in TTS	Diagnostic/ Differential Value	References
***VALIDATED BIOMARKERS***				
**Troponin**	Myocardial injury	- Elevated on admission - Mild–moderate increase during hospitalization	Lower levels in TTS compared to ACS	[7,70]
**Troponin I + LVEF**			Useful to distinguish TTS from STEMI	[71]
**Creatine Kinase**	Myocardial injury	Slight increase	Useful to distinguish TTS from MI	[7]
**NT-proBNP**	Myocardial injury	- Markedly elevated - Peak approximately 24–48 h after symptom onset	Higher in TTS than ACS High concentration closely correlates with the extent of ventricular wall motion abnormality	[7,18,40]
***EMERGING CIRCULATING PROTEINS***				
**Platelet CD62P Expression**	Platelet activity marker	Low levels	Significantly lower in TTS compared to MI	[75]
**IL-6 Plasma Levels**	Inflammatory mediator	Low levels	Significantly lower in TTS compared to MI	[75]
**IL-7 Plasma Levels**	Inflammatory mediator	Elevated at 2–4 days after hospital admission	Significantly elevated in TTS compared to MI	[75]
**GDF-15**	Stress responsive cytokine	markedly high but transient elevation	Significantly elevated on admission compared to STEMI Especially high concentrations in cases of biventricular ballooning Admission levels strongly predict adverse clinical outcomes	[76]
***EMERGING CIRCULATING miRNAs***				
**miR-133a**	Myocardial injury	Sharply increased	Significantly correlated with cardiac troponin T levels in serum Correlated with the onset of myocardial damage	[79]
**miR-16, miR-26a, miR-1, and miR-133a**	Stress- and depression- related miRNAs	Upregulation	Signature which robust distinguish TTS from STEMI	[80]
**miR-125a-5p**	Microvascular spasm	Downregulation and increased plasma levels of its target, ET-1	Tendency that may distinguish TTS patients from healthy subjects	[80]

ACS: acute coronary syndrome; ET-1: endothelin-1; GDF-15: growth/differentiation factor 15; MI: myocardial infarction; miRNAs: microRNAs; NT-proBNP: N-terminal pro-B-type natriuretic peptide; STEMI: ST-segment elevation myocardial infarction; TTS: Takotsubo syndrome.

## 5. Extracellular Vesicles, Takotsubo Syndrome, and Oxidative Stress

Recently circulating EVs have emerged as potential diagnostic and prognostic tools in different clinical settings, including cardiovascular conditions [30,31,84,85,86,87,88,89].

EVs are lipid bilayer membranous particles released from the surface of different cell types under both physiological and pathological conditions. According to the Minimal Information for Studies of Extracellular Vesicles guidelines, EVs can be classified based on their physical characteristics, for example, size in “small EV” (“sEV”; <100 nm or <200 nm) and “medium/large EV” (“m/L EV”; >200 nm) or density (low, middle, and high, with each range defined) but also by biochemical features, cell culture conditions, or cell of origin [28].

In the last decade, EVs have been increasingly recognized as potential significant autocrine and paracrine communicators. Intercellular communication and coordinators among different types of cardiac cell are pivotal for the integrity and proper function of the organ [90].

EVs act as reservoirs containing a multitude of functional biomolecules, including lipids, proteins, amino-acids, mRNAs, and miRNAs, which mirror the composition of the donor cell and reflect the environmental conditions sensed by their cell of origin [27]. EVs represent promising diagnostic and prognostic biomarkers not only because of their molecular cargo but also due to the expression of specific surface proteins on their membranes. EVs can be considered vector signalosomes due to their variable protein content, which can also determine their functionality in several ways [27]. Indeed, the biodistribution and binding of EVs to target cells or the extracellular matrix rely on surface-exposed receptors and ligands. Afterwards, EVs can activate intracellular signaling pathways either through simple interaction with surface receptors or ligands on target cells or through internalization [90].

According to the intrinsic miRNA properties, including stability and resistance to RNAse degradation, a considerable number of investigations have been focused on evaluating EV–miRNA content for biomarker discovery in cardiac diseases. Indeed, Kubawara et al. [79] demonstrated that serum levels of miR-133a were significantly elevated in TTS patients and can be used as a biomarker for myocardial injury, also studying its localization within exosomes and its release after Ca^2+^ stimulation. In addition, miR-133a has been extensively studied, as several molecular targets have been validated in the context of cardiovascular diseases. Izarra et al. [91] showed that miR-133a overexpression protects adult cardiac progenitor cells from oxidative stress-induced cell death by inhibiting pro-apoptotic genes such as Bim and Bmf. It was further evidenced that secreted miR-133a was mainly incorporated into the exosomal fraction derived from cardiac progenitor cells [91]. Furthermore, miR-133a modulates cardiac hypertrophic signaling by targeting: RhoA, a GDP-GTP exchange protein regulating cardiac hypertrophy, and Cdc42, a signal transduction kinase implicated in hypertrophy [92]. miR-133a can also attenuate cardiac hypertrophy by repressing the expression of serum response factor (SRF) and cyclin D2; other targets include the cardiogenic transcription factor and myocyte enhancer factor 2 (MEF2), as well as serum- and glucocorticoid-responsive kinase-1 (SGK1) and insulin-like growth factor-1 receptor (IGF-1R) [93]. These signaling networks help to elucidate both the functional roles in cardiac disease and the potential clinical application as a biomarker of EV-encapsulated miR-133a.

Fan et al. [94] first demonstrated that exosomal miR-126-3p, derived from endothelial cells, induces ion channel dysfunction by targeting the regulator of G-protein signaling 3 (RGS3) signaling in cardiomyocytes. Regulators of G-protein signaling proteins, including RGS3, are known to suppress the activity of G-proteins. Since G-proteins are functionally linked to adrenoceptors, the inhibition of RGS3 by miR-126-3p may potentiate or simulate catecholamine signaling by reducing RGS3 effect on G-protein activity, thereby amplifying adrenoceptor/G-protein-dependent effects [94]. Moreover, the serum levels of miR-126-3p were measured in patients with acute and reversed TTS. The results showed that miR-126-3p, miR-26a, and miR-133a, as well as epinephrine, were significantly increased in patients with acute TTS compared to healthy donors [94]. Interestingly, a significant miRNA and epinephrine reversion was detected in patients in the healing phase of TTS, supporting the role of miR-126-3p and epinephrine in Takotsubo cardiomyopathy pathogenesis [94].

Recently, Nejat et al. [95] developed and validated a novel method for the extraction and characterization of EVs from an ex vivo model of TTS using isolated rat hearts, showing distinct protein profiles in EVs from apical segments.

Notably, Zulfaj et al. [96] performed a global proteomic analysis of EVs isolated from 24 h TTS-induced rat hearts, revealing a distinct protein profile in EVs from the apical segment of TTS hearts but not in the basal segments. Furthermore, functional enrichment analysis of the differential expressed proteins highlighted a marked modulation of biological processes, including metabolic lipid processes, inflammation, complement activation, and extracellular matrix reorganization [96]. On the other hand, biological processes related to mitochondrial ATP synthesis coupled electron transport and muscle contraction have been found to be downregulated [96]. Subsequently, Zulfaj et al. [97] conducted a more in-depth analysis, showing the proteomic characterization of EVs in the context of TTS rat hearts across its natural course for the first time. The differential expressed proteins that emerged from the analysis are biologically associated with immune response, tissue repair, survival signaling, metabolism, and ROS detoxification [97]. This broad catalog of differentially expressed proteins identifies potential therapeutic targets and diagnostic biomarkers.

It is well-demonstrated that, under oxidative stress stimuli, EV release increases [98]. Although direct studies focused on the analysis of EVs in oxidative stress in TTS patients are currently limited, as mediators of cell-to-cell communication, EVs are also involved in the pathophysiology of oxidative stress-related diseases [98]. General evidence supports the idea that EV release and cargo composition are strongly regulated by oxidative stress [98]. Furthermore, antioxidants, oxidized molecules, or redox-active proteins can be carried by EVs and can modulate the oxidative status of target cells, exerting both beneficial and harmful effects [98]. Under oxidative stress conditions, changes in phosphorylation levels were observed in EV protein cargo involved in cell proliferation, survival, and energy metabolism pathways [99]. Oxidatively stressed cells and EVs displayed a downregulation of pro-survival proteins and an enhancement of pro-apoptotic proteins, and therefore cells subjected to oxidative stress might modulate these signaling pathways via EV molecules [98,99]. In addition to RNA and proteins, oxidized lipids generated from the peroxidation of cell membrane phospholipids are conveyed by EVs released into an oxidative stress environment. EV-mediated transfer of lipid peroxidation products seems to exert biologically relevant effects on target cells [98]. Furthermore, EVs can convey both antioxidants, thus acting as ROS scavengers, and enzymes that, in turn, are involved in ROS production [100].

In recent years, it has been widely demonstrated that EVs may be mediators of oxidative stress, inflammation, and progressive endothelial cell dysfunction in cardiovascular system, thus contributing to cardiovascular disease [98]. Given that oxidative stress is implicated in the myocardial response to catecholamine overload in stress-induced cardiomyopathy, it may be reasonable to hypothesize a plausible link between redox dysregulation and EV biology in TTS.

**Figure 3:**
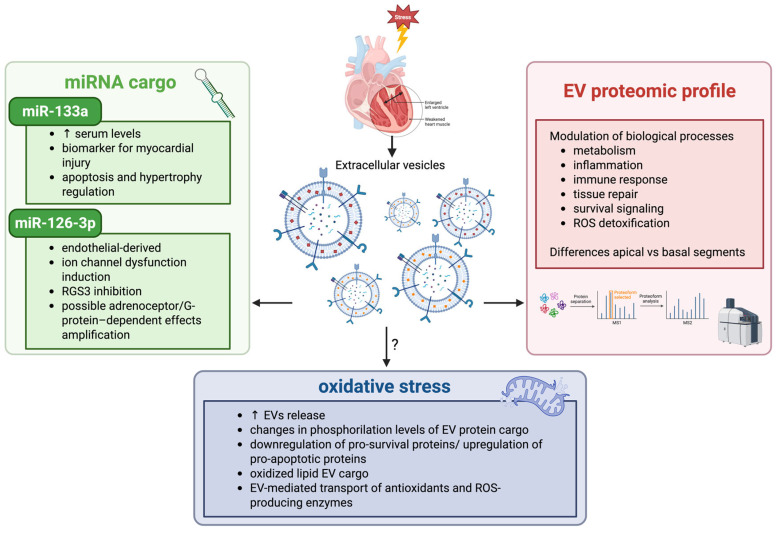
Schematic illustration of the mechanisms that link EVs, TTS, and oxidative stress. TTS is characterized by stress-induced myocardial dysfunction, which in turn causes the release of EVs. EVs may carry miRNAs such as miR-133a, a circulating biomarker of myocardial damage, and miR-126-3p, an endothelial-derived exosomal miRNA involved in altered G-protein signaling. Proteomic studies of EVs in experimental models of TTS reveal region-specific molecular signatures with modulation of biological processes, including metabolism, inflammation, tissue repair, and ROS detoxification. Oxidative stress may further influence EV release and cargo composition, contributing to myocardial functional impairment. EVs: extracellular vesicles; RGS3: regulator of G-protein signaling 3; ROS: reactive oxygen species; ↑: increase.

Although further studies are needed, oxidative stress appears to play a pivotal role in the development of TTS, and EVs could represent an interesting tool for the diagnosis and prognosis of TTS cardiomyopathy (Figure 3).

## 6. Conclusions and Future Perspectives

TTS remains a not fully elucidated form of acute cardiac dysfunction. It is characterized by a transient impairment of left ventricular performance driven by a complex interaction of neurohumoral activation, redox imbalance, mitochondrial alterations, inflammatory responses, and vascular dysfunction [1,2]. Despite growing clinical awareness, the absence of reliable and disease-specific biomarkers continues to hamper timely diagnosis and individualized patient management.

In this context, EVs represent a promising and biologically plausible source of diagnostic and prognostic information [27,29,30]. By reflecting the molecular fingerprint of their cells of origin, EVs integrate signals related to oxidative stress, myocardial injury, vascular dysfunction, and systemic inflammation. These processes are central to TTS pathophysiology [44,47]. The ability of EVs to transport redox-sensitive microRNAs, proteins, lipids, and antioxidant or pro-oxidant enzymes positions them as attractive candidates for capturing both the acute phase and recovery dynamics of the syndrome [77,78]. Importantly, EV-associated cargo may provide greater specificity and stability than conventional circulating biomarkers, offering insights into disease mechanisms rather than merely downstream injury.

Future research should focus on large, well-characterized cohorts to validate EV-derived signatures associated with TTS onset, severity, complications, and long-term outcomes. Standardization of EV isolation, characterization, and analytical methodologies will be essential to ensure reproducibility and clinical applicability. Integrating EV profiling with clinical data, imaging findings, and other omics approaches may further enhance diagnostic accuracy and uncover novel mechanistic pathways involved in stress-induced cardiomyopathy.

From a translational perspective, EV-based biomarkers hold potential not only for diagnosis and prognosis but also as instruments for supporting personalized therapeutic decision-making. In the longer term, targeting EV-mediated redox signaling pathways may open new avenues for intervention aimed at mitigating oxidative stress, improving endothelial function, and reducing susceptibility to recurrence. Overall, advancing our understanding of EVs in the context of oxidative stress and TTS may contribute significantly to bridging the gap between pathophysiological insights and precision cardiovascular care.

## Figures and Tables

**Table 1:** Diagnostic techniques useful in clinical practice to make differential diagnoses and identify TTS.

Diagnostic Techniques	Key Findings in TTS	Differential Diagnosis	References
**Electrocardiogram**	ST-segment elevation T-wave inversion ST-segment depression Left bundle branch block	ACS	[7,40]
**InterTAK Diagnostic Score**	Female sex Emotional trigger Physical trigger Absence of ST-segment depression Psychiatric disorders Neurologic disorders QTc prolongation	ACS	[56]
**Endomyocardial Biopsy**	Myocardial inflammation	Myocarditis	[58,59]
**Circulating Inflammatory Cytokines**	Tumor necrosis factor- α, IL-6, IL-1β, and catecholamines	Myocarditis	[60,61]
**Coronary Angiography and Left Ventriculography**	Obstructive coronary disease exclusion Identification of wall motion abnormalities: typical apical ballooning pattern but also midventricular, basal, and focal variants	ACS	[40,62]
**Echocardiography**	LV dysfunction Detection of acute TTS complications: LV outflow tract obstruction, mitral regurgitation, thrombus formation, and ventricular rupture	ischaemic cardiomyopathy	[63]
**Cardiac Magnetic Resonance Imaging**	Useful in subacute evaluation of TTS Evaluation of RV and LV function Assessment of additional complications High-resolution characterization of myocardial tissue (edema, inflammation, necrosis/fibrosis) Absence of LGE Gold standard for follow-up to confirm reversibility within 3–6 months	Acute myocardial infarction and myocarditis	[40,62,67]
**Cardiac Computed Tomography**	Coronary artery anatomy Regional LV contraction	CAD	[68]
**SPECT/PET**	Assessment of myocardial perfusion, metabolism, and innervation	ACS	[40]

ACS: acute coronary syndrome; CAD: coronary artery disease; IL-1β: interleukin-β; IL-6: interleukin-6; LGE: late gadolinium enhancement; LV: left ventricular; RV: right ventricular; TTS: Takotsubo syndrome.

## Data Availability

No new data were created or analyzed in this study. The original contributions presented in this study are included in the article. Further inquiries can be directed to the corresponding author.

## Abbreviations

The following abbreviations are used in this manuscript:

ACS	acute coronary syndrome
BH4	tetrahydrobiopterin
BNP	B-type natriuretic peptide
CACNB1	L-type calcium channel Cavβ subunit
CAT	catalase
CK-MB	creatine kinase-myocardial band
CRP	C-reactive protein
CT	computed tomography
CVDs	cardiovascular diseases
ECG	electrocardiogram
EF	ejection fraction
eNOS	nitric oxide synthase
ET-1	endothelin-1
EVs	extracellular vesicles
GDF-15	growth/differentiation factor-15
GNB1	G-protein subunit Gβ
GPx	glutathione peroxidase
IGF-1R	insulin-like growth factor-1 receptor
IL-1 β	interleukin-1 β
IL-6	interleukin-6
LGE	late gadolinium enhancement
LV	left ventricular
LVOTO	left ventricular outflow tract obstruction
LVEF	left ventricular ejection fraction
MAPKs	mitogen-activated protein kinases
microRNA	miRNA
MEF2	myocyte enhancer factor 2
MRI	magnetic resonance imaging
NF-κB	nuclear factor kappa-light-chain-enhancer of activated B cells
NO	nitric oxide
NSTEMI	non-ST-elevation myocardial infarction
NT-proBNP	N-terminal pro-B-type natriuretic peptide
PET	positron emission tomography
RGS3	regulator of G-protein signaling 3
RGS4	regulator of G-protein signaling 4
ROS	reactive oxygen species
RV	right ventricular
SERCA2a	sarco/endoplasmic reticulum Ca^2+^-ATPase 2a
SGK1	serum- and glucocorticoid-responsive kinase 1
SOD	superoxide dismutase
SPECT	single-photon emission computed tomography
SRF	serum response factor
STEMI	ST-segment elevation myocardial infarction
TnI	troponin I
TTS	Takotsubo syndrome
